# Physiological impacts of atmospheric pollution: Effects of environmental air pollution on exercise

**DOI:** 10.14814/phy2.16005

**Published:** 2024-04-11

**Authors:** Michael Stephen Koehle

**Affiliations:** ^1^ School of Kinesiology University of British Columbia Vancouver British Columbia Canada; ^2^ Division of Sport & Exercise Medicine University of British Columbia Vancouver British Columbia Canada; ^3^ Canadian Sport Institute – Pacific Victoria British Columbia Canada; ^4^ Athletics Canada Ottawa Ontario Canada

**Keywords:** acclimation, air pollution, exercise, masks, performance, physical activity

## Abstract

In this review, we discuss some of the recent advances in our understanding of the physiology of the air pollution and exercise. The key areas covered include the effect of exercise intensity, the effects of pre‐exposure to air pollution, acclimation to air pollution, and the utility of masks during exercise. Although higher intensity exercise leads to an increase in the inhaled dose of pollutants for a given distance traveled, the acute effects of (diesel exhaust) air pollution do not appear to be more pronounced. Second, exposure to air pollution outside of exercise bouts seems to have an effect on exercise response, although little research has examined this relationship. Third, humans appear to have an ability to acclimate to ground level ozone, but not other pollutants. And finally, masks may have beneficial effects on certain outcomes at low intensity exercise in pollution with significant levels of particles, but more study is required in realistic conditions.

## 1 INTRODUCTION

The negative effects of air pollution on health are clear. Chronic exposure to air pollution increases risk of mortality (Andersen et al., 2015), dementia (Raichlen et al., 2022), Type 2 diabetes (Yang et al., 2020), lung cancer (Andersen et al., 2015), heart disease (Giles & Koehle, 2014), and chronic obstructive pulmonary disease (Giles & Koehle, 2014). Likewise, the positive effects of exercise in terms of prevention and management of non‐communicable disease (NCD) are so significant that physical inactivity has been listed as a risk factor for NCD (Katzmarzyk et al., 2022). However, some of the characteristics of exercise, such as increased ventilation and pulmonary diffusing capacity, also increase the inhaled dose of air pollutants and the potential for adverse effects (Daigle et al., 2003). Squaring this circle is a challenge for physiologists, policy makers, and those who prescribe and counsel exercise.

The purpose of this review is to highlight some of the recent advances in the physiology of the air pollution and exercise interaction. The key areas that will be covered will include the effects of pre‐exposure to air pollution, acclimation to air pollution, the effect of exercise intensity, including the performance effects of air pollution. Finally, we will discuss the potential of masks during exercise in poor air quality. In‐depth summaries of the acute effects of air pollution and exercise (Hung et al., 2021), and strategies to mitigate those effects have recently been published (Hung et al., 2023) elsewhere.

## 2 BACKGROUND

**FIGURE 1:**
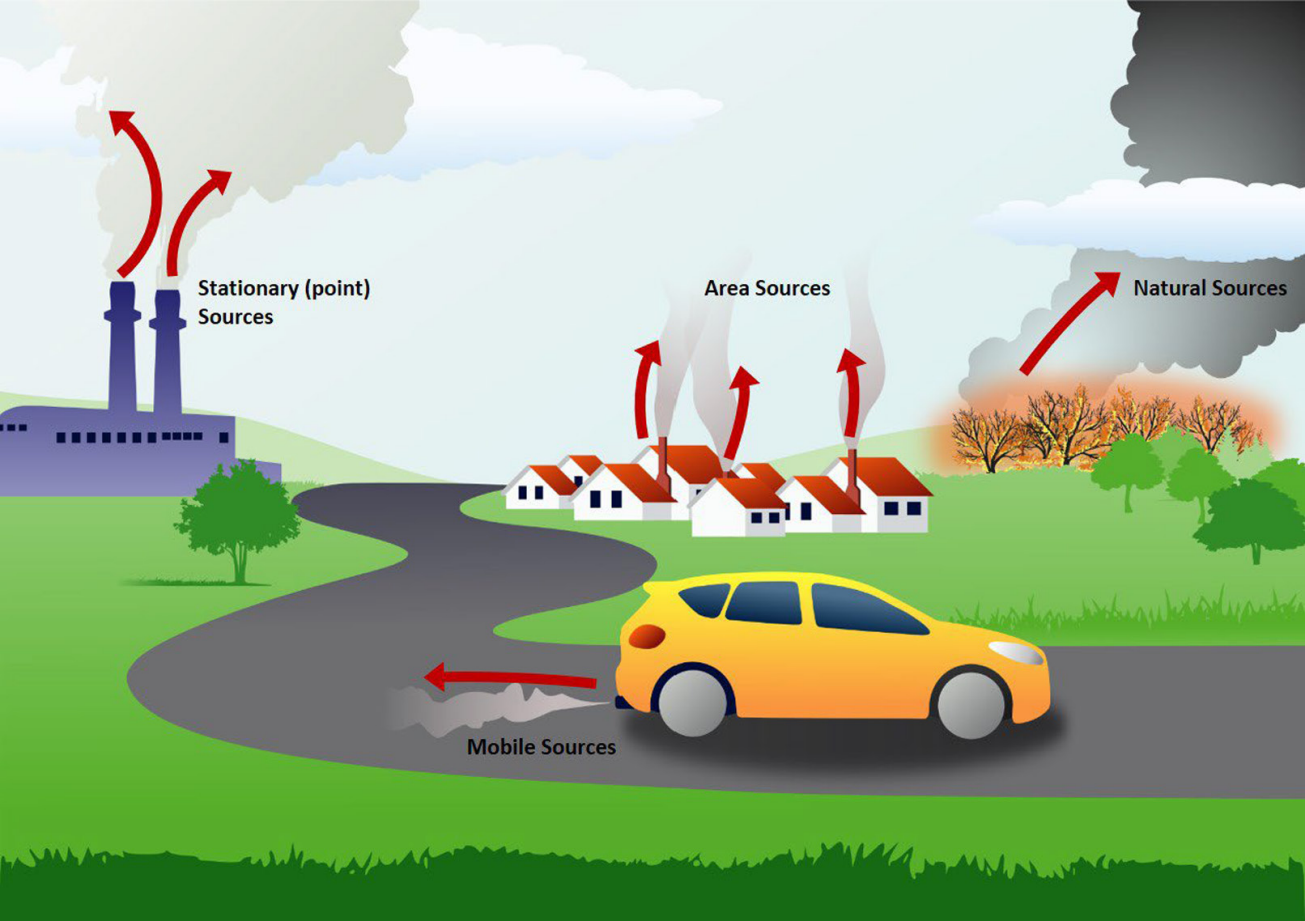
Sources of air pollution (Courtesy of Environmental Protection Agency). https://www.epa.gov/ecobox/epa‐ecobox‐tools‐exposure‐pathways‐air.

**FIGURE 2:**
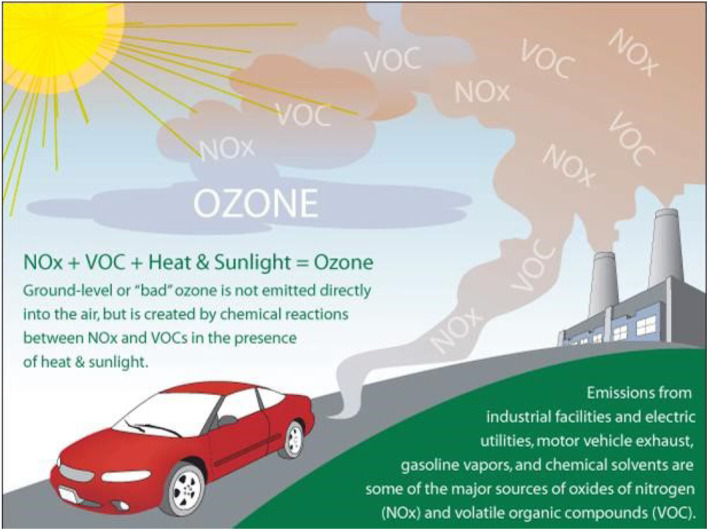
Formation of ground‐level ozone via the reaction of oxides of nitrogen and volatile organic compounds with UV radiation (Courtesy of Environmental Protection Agency). https://www.epa.gov/ground‐level‐ozone‐pollution/ground‐level‐ozone‐basics.

Air pollution comes from a variety of primary sources, including natural sources (such as dust and pollen), stationary sources (such as industrial emissions), mobile sources (e.g., car exhaust). Furthermore, secondary pollutants (e.g., ground level ozone) can be created from the modification of primary pollutants (Figure 1). When considering health and performance effects, we must not treat air pollution as a single homogenous stressor. In fact, it is a constantly changing mixture of gases, liquids, and solids that varies depending on a variety of factors, including the source, location, time of day, season, and weather. Thus, whenever considering air pollution's effects we need to consider the specific recipe of air pollution for any given situation. For example, on hot sunny days, ultraviolet radiation from the sun converts primary pollutants such as volatile organic compounds (VOCs) and oxides of nitrogen (NOx) into ozone (O_3_), a highly reactive secondary air pollutant with profound respiratory effects (Figure 2). Conversely, in areas affected by wildfires, a high burden of particles (such as black carbon) represents the predominant constituent, often with relatively lower levels of ozone due to lower levels of UV radiation and substrate. Particles are often subcategorized according to aerodynamic diameter with PM_10_ representing particles ≤10 μm in diameter, PM_2.5_ referring to those ≤2.5 μm and particles ≤0.1 μm referred to as ultrafine particles (UFP).

**FIGURE 3:**
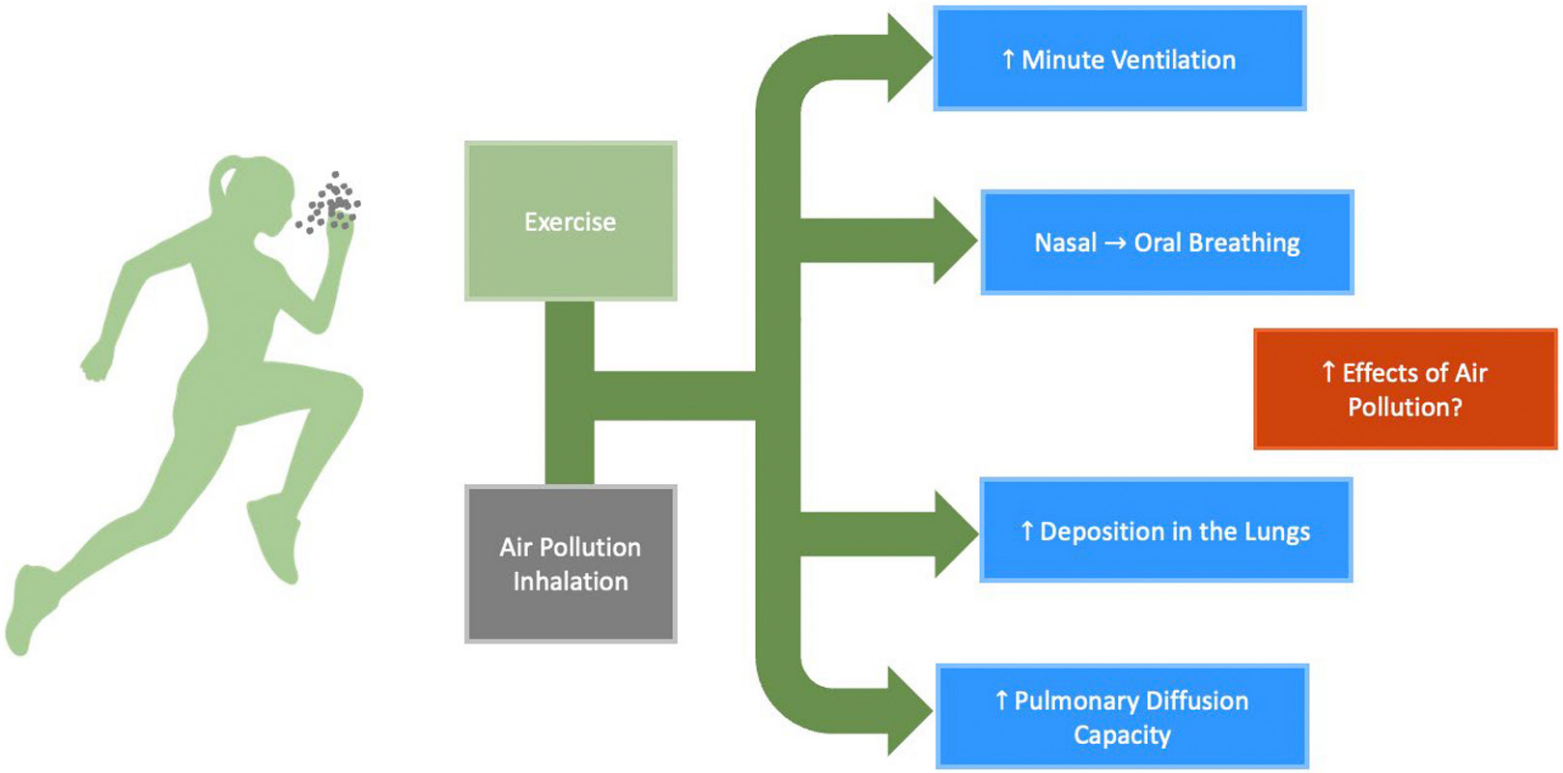
With Permission by Andy Hung, adapted from Giles and Koehle (2014).

When we exercise in air pollution, several factors have the potential to exacerbate the adverse consequences of air pollution (Figure 3). Specifically, exercise leads to an acute increase in ventilation, oral breathing (as compared to nasal breathing) (Daigle et al., 2003), tidal volume and the ability of gases to diffuse from the lung to the bloodstream (i.e., diffusing capacity) (Brook et al., 2010; Qvarfordt et al., 2022). Modeling demonstrates that the rate of particle deposition in the lung increases with intensity and the total dose increases with exercise duration (Cruz et al., 2021). This subsequent increase in dose of air pollutants has the potential for greater adverse effects (Giles & Koehle, 2014). Thus, physiologists are in a quandary when advising individuals and groups around exercise in air pollution. The beneficial effects of exercise are legion, but the theoretical potential for an increase in dose and effects from the pollution requires consideration.

## 3 CURRENT KNOWLEDGE ON AIR POLLUTION AND EXERCISE

Fortunately, the interaction between air pollution and physical activity is increasingly being investigated. When we describe the combined effects of air pollution and exercise, we need to consider the perspective through which we examine these effects. Both the *health* and the *performance* effects are of interest to physiologists, especially those working in high‐performance sport. Furthermore, we need to consider the acute effects (i.e., during and immediately after exercise in air pollution) and the chronic effects (i.e., the long‐term effects of an active lifestyle when chronically exposed to poor air quality). The methods of study of each of these lenses differ (Table 1), with relative strengths and weaknesses of each design. When evaluating any study, one must first determine the lens that is being used, and the appropriateness of the methodology.

**TABLE 1:** The research and outcomes matrix for air pollution and exercise.

Timeline	Health	Performance
Acute	Laboratory exposure studies, ambient pollution exposure studies	Laboratory exposure studies, observational studies
Chronic	Epidemiological studies	Observational (cohort) studies

In general, epidemiological studies are best for studying the chronic health effects of an active lifestyle in a polluted environment, but they generally lack blinding and randomization and are therefore prone to confounders. Epidemiological studies have been used to investigate a variety of health outcomes including mortality (Andersen et al., 2015; Sun et al., 2019) from a variety of causes, as well as incidences of type 2 diabetes (Guo et al., 2021) and dementia (Raichlen et al., 2022). In general, they show that the positive chronic health effects of physical activity persist, especially when pollution levels are low and moderate (Andersen et al., 2015; Guo et al., 2021; Raichlen et al., 2022; Sun et al., 2019).

By contrast, the evidence for acute health effects from air pollution comes from a mix of laboratory exposure studies using pollution generated in a chamber, or ambient studies, where the participants exercise outdoors in a polluted environment. The evidence for acute adverse health effects while exercising is most convincing with ground‐level ozone (Hung et al., 2021). The majority of these studies show worsening of symptoms (such as cough and chest tightness) and a degradation of pulmonary function following exercise in high levels of ozone. By contrast, from a health point of view, the acute effects of exercise in traffic‐related air pollution (TRAP) and diesel exhaust appear to be more equivocal (Hung et al., 2021) with a greater proportion of research showing no effect or even a positive effect of exercise in air pollution. A similar situation is present for acute performance effects. Ground‐level ozone exposure during exercise consistently decreases peak oxygen consumption (Hung et al., 2021) and competition performance (Mullins, 2018). However, the research examining the performance effects of TRAP (Silveira et al., 2022) and diesel exhaust (Giles et al., 2014) have not consistently shown acute detrimental effects from either pollutant.

To summarize the current state of knowledge on physical activity, exercise, and air pollution, the general conclusion is that habitual physical activity yields health benefits over the long term, even in areas with low to moderate air pollution. However, in the short term, engaging in exercise in environments with high levels of ozone can result in acute health and performance effects.

## 4 EXERCISE INTENSITY IN AIR POLLUTION

A question often asked of physiologists regarding air pollution and exercise is: “Is it better to do a long, easy workout when the air quality is poor, or should one do a shorter more intense bout?”. Put another way: “If I am riding my bike to work on a polluted day, should I go as fast as possible, or really take it easy, even if it means I will be exposed the pollution for longer?” Given that the above‐mentioned characteristics of exercise (increased ventilation, metabolism, diffusing capacity, and oral breathing) should be more pronounced during high‐intensity exercise, one would expect that the effects of air pollution during exercise would be exacerbated during higher intensity exercise. The potential for an increased dose of pollutants with higher intensity exercise is not only theoretical, but has been demonstrated empirically (Marmett et al., 2021). For the same distance covered, participants breathed more, and inhaled more particles in the high intensity condition. Unfortunately, very little work has examined this question in a rigorous manner. Using a double‐blind crossover design, Giles et al. (2014, 2018, 2019) asked 18 healthy males to perform rest, low‐intensity exercise, or high‐intensity exercise in both a clean air and diesel exhaust condition. They looked at a variety of outcomes, including pulmonary function, flow‐mediated dilatation, inflammation, oxygen consumption, and perceived exertion. Across all these parameters, there were minimal differences between the diesel exhaust and clean air condition, regardless of intensity. The investigators only noted an increase in ventilation and oxygen consumption in the diesel condition following low‐ but not high‐intensity exercise. The lack of exacerbation of a pollution effect in high‐intensity exercise was surprising and may relate to a number of factors. Primarily, despite the very high dose of diesel exhaust, there were minimal differences between the diesel and clean air condition (at all intensities). Fortunately, this minimal effect of diesel exhaust has already been replicated with a similar exposure and design, but in participants with asthma (Koch et al., 2021, 2020). Since the work rate was controlled, the increased oxygen consumption could relate to a decrease in efficiency, possibly through increased respiratory work, although why this was not evident with high intensity is not clear. Instead, the authors attribute these findings to the profound stimulus of exercise obscuring the more modest diesel exhaust effect at all intensities. Although this is the only group of studies to comprehensively look at intensity in air pollution, it only looked at effects immediately after exercise and in the 2 h post‐exercise. So, although there were no observed short‐term effects of an increased exercise intensity, the medium and longer term effects were not assessed. Also, they used diesel exhaust as the air pollution stimulus, so these findings cannot be extrapolated to other types of air pollution (such as TRAP and ozone exposure). In summary, although preliminary work has not shown a worsening with increased exercise intensity, more research (especially with different types of exposures) is necessary across a broader range of conditions before making definitive recommendations.

## 5 PRE‐EXPOSURE TO AIR POLLUTION

Although the focus of this paper is on air pollution exposure during exercise, individuals are rarely exposed to pollution only at the time of exercise, without some exposure occurring also during the non‐exercise time. For example, air pollution can be experienced on the way to and from training and competition, and likewise, ambient air pollution can penetrate all but the most controlled indoor environments. As such, during periods of poor air quality, individuals are typically exposed to some levels of polluted air throughout the day. What is the effect of resting exposure to air pollution on subsequent exercise performance? We are aware of two studies that are relevant to this question. In a laboratory exposure study, Giles et al. (2012) exposed healthy individuals to diesel exhaust at (300 μg/m^3^) for 60 min while at rest. This resting exposure was followed by a 20 km cycling time trial in clean air. The investigators found that following the diesel exhaust exposure there was a relative increase in heart rate during exercise as compared to the clean air condition. Furthermore, with the diesel pre‐exposure, the normal exercise‐induced bronchodilation that occurs with an exercise bout was absent. The authors concluded that these subtle differences were as a result of the pre‐exposure to air pollution. In an observational study using ambient air pollution in three cities over three seasons, the investigators monitored total air pollution exposure (as represented by black carbon) and physical activity in 115 healthy individuals (Laeremans et a., 2018a). They concluded that there was a beneficial effect of physical activity on measures of lung function when daily black carbon exposure was low, but that at higher levels of black carbon, this beneficial effect was decreased. With the design of the study, the pollution exposure during exercise was not considered, only the total daily exposure. Thus, at minimum both of these studies indicate that more research is needed to assess the effect of non‐exercise pollution exposure on the interaction between air pollution and exercise.

## 6 ACCLIMATION

Interestingly, the focus of pollution and exercise research has varied based on the most prominent ambient air pollutants. Much of the research in the 1980's was focused on ground‐level ozone, and one of the areas of investigation was the potential of humans to acclimate to ozone over successive days.

As mentioned, ground‐level ozone is the pollutant with the clearest evidence for harmful effects during and after exercise (Hung et al., 2021). Multiple studies have shown both health (i.e., lung function) and performance effects following acute ozone exposure during exercise. However, studies that have used successive ozone exposures (Foxcroft & Adams, 1986; Hackney et al., 1977) to look at lung function effects have shown an initial drop that at least partially reversed after the third day of exposure. However, more recently, Mullins (2018) looked at the question of adaptation with a completely different approach that focused on performance. Instead of direct laboratory exposures, they compared 668,277 performances at approximately 1700 intercollegiate track meets. Mullins compared home and competition ambient ozone levels and looked at the decrement in performance at a track meet attributable to the ozone level. The author's key finding was that individuals from a high ozone environment had less of a performance decrement when competing at a high ozone track meet. He concluded that this effect might be a result of an acclimatization to ozone (Mullins, 2018).

Although there seems to be some evidence of acclimatization to both the health and performance effects of ozone, there is not yet evidence of such an effect in TRAP or PM (Hung et al., 2023). One study has looked at repeated exposures to very high levels of NO_2_ (Blomberg et al., 1999) over 4 days, noting an initial drop in FEV_1_ and FVC after the first exposure that was at least partially mitigated on subsequent days. The NO_2_ levels to which the participants were exposed were particularly high and neither the exposures nor the outcome testing involved exercise, so definite conclusions cannot yet be made. Likewise, the evidence for adaptation to particulate matter exposure during exercise is not compelling.

To summarize, there are a variety of lines of evidence in favor of an ability to adapt to ozone in the context of exercise. Thus, there is a potential for deliberate ozone acclimation as a potential strategy for reducing its adverse effects during sport and exercise (Sandford et al., 2021). Since high ozone levels occur concomitantly with high temperatures, a specific ambient heat acclimation plan can often expose individuals (and hence acclimate them) to ozone at the same time. Nevertheless, applied research into the safety, efficacy and optimal protocols for ozone acclimation is required before specific recommendations can be made.

## 7 MASKS

Particle filtration masks have the potential to reduce inhaled particles, and thus the particle‐mediated effects of air pollution. Surprisingly little research has investigated the effects of mask‐wearing during exercise in air pollution, and many of the studies are neither controlled nor blinded. For example, in a meta‐analysis of seven other studies that were incompletely blinded, and mostly in clinical populations, mask wearing led to a small decrease in systolic and mean arterial pressure (Han et al., 2021).

Fortunately, Guan et al. (2018) did conduct a double‐blinded crossover RCT of the short‐term health effects of facemasks in ambient air pollution. Fifteen healthy adults walked along a roadside for 2 h during particularly poor air quality in Beijing (mean PM_2.5_ of 204 ug/m^3^). They found that the masks ranged from 48% to 74% efficiency in terms of reducing particle count in real‐world conditions. Furthermore, they noted that the increases in interleukin‐1α, interleukin‐1β, interleukin‐6 and exhaled nitric oxide resulting from air pollution were partially attenuated in the filtration mask condition. There were no beneficial effects of the mask on arterial pulse wave analysis outcomes. It is important to note that of the published studies examining mask wearing and activity, there are none that include exercise of greater intensity than walking. Furthermore, in an oft‐cited study of the beneficial effects of mask wearing on blood pressure and heart rate variability (Langrish et al., 2009), not only were the participants unblinded, but they were required to wear the masks for 24 h on the day prior to, and the day of their exercise bout. Thus, it is impossible to determine whether the benefit from the mask came from the walking exposure or the 2 days of mask wearing around the walking bout (See discussion of pollution pre‐exposure above).

For exercisers to consider wearing a mask during exercise, the issue of tolerability and the effect that masks have on exercise performance are particularly relevant. With the recent pandemic, there has been much interest in recent years around the effects of mask‐wearing on exercise performance. A recent meta‐analysis looked at psychological and physiological parameters resulting from mask‐wearing, with a subgroup analysis for the relevant N95/FFP2 type masks (Zheng et al., 2023). The authors noted that with these types of masks, there were clear increases in dyspnea, fatigue and exercise performance in the N95/FFP2 subgroup. These outcomes relate to both the increase in resistance provided by the mask, as well as the increase in dead space, leading to an increase in end‐tidal carbon dioxide.

Thus, with an absence of robust research looking directly at mask wearing during exercise in a polluted environment, and the psychological and physiological decrements associated with their use during moderate‐to‐vigorous physical activity, mask wearing during sport and exercise has not been widely recommended (Hung et al., 2023). However, given the potential for pre‐exposure to air pollution prior to exercise to have an effect (Giles et al., 2012; Laeremans et al., 2018b; Langrish et al., 2009), it is reasonable for individuals to consider wearing a well‐fitting N95/FFP2 mask on the way to and from exercise, when particulate levels are high (Hung et al., 2023).

## 8 CONCLUSION

Although the beneficial effects of physical activity and the negative effects of air pollution are reasonably well‐defined, their interaction remains a subject of investigation due to the increase in inhaled dose of pollutants during exercise. The effect of exercise intensity on dose and health and performance effects requires more investigation, but recent work in diesel exhaust shows surprisingly minimal acute health and performance effects and no worsening in higher intensity.

In contrast, exposure to air pollution prior to exercise challenge reveals subtle yet significant effects, such as altered heart rate during subsequent exercise and potential reductions in the beneficial effects of physical activity under higher pollution levels. Meanwhile, the potential for acclimation to ozone has been demonstrated through a variety of methodologies. This acclimation may have promise in developing adaptive strategies to ozone prior to exposure, however more research is required.

And finally, masks present a potentially helpful tool, but research on their effectiveness during exercise in real‐world conditions is limited. While some studies indicate a reduction in particle count and attenuation of certain health markers, their tolerability, their effect on exercise performance and their utility in exercise of a higher intensity than walking require further study.

## Ethics statement

No funding sources to declare.

## Data Availability

Data sharing is not applicable to this article as no new data were created or analyzed in this study.
